# Coronary endothelial function is better in healthy premenopausal women than in healthy older postmenopausal women and men

**DOI:** 10.1371/journal.pone.0186448

**Published:** 2017-10-26

**Authors:** Lena Mathews, Micaela Iantorno, Michael Schär, Gabriele Bonanno, Gary Gerstenblith, Robert G. Weiss, Allison G. Hays

**Affiliations:** 1 Department of Medicine, Division of Cardiology, Johns Hopkins School of Medicine, Baltimore, Maryland, United States of America; 2 Department of Radiology, Division of Magnetic Resonance Research, Johns Hopkins School of Medicine, Baltimore, Maryland, United States of America; University of Bologna, ITALY

## Abstract

**Background:**

Premenopausal women have fewer cardiovascular disease (CVD) events than postmenopausal women and age-matched men, but the reasons are not fully understood. Coronary endothelial function (CEF), a barometer of coronary vascular health, promises important insights into age and sex differences in atherosclerotic CVD risk, but has not been well characterized in healthy individuals because of the invasive nature of conventional CEF measurements. Recently developed magnetic resonance imaging (MRI) methods were used to quantify CEF (coronary area and flow changes in response to isometric handgrip exercise (IHE), an endothelial-dependent stressor) to test the hypothesis that healthy women have better CEF compared to men particularly at a younger age.

**Methods:**

The study participants were 50 healthy women and men with no history of coronary artery disease (CAD) or traditional CV risk factors and Agatston coronary calcium score (on prior CT) <10 for those ≥ 50 years. Coronary cross-sectional area (CSA) measurements and flow-velocity encoded images (CBF) were obtained at baseline and during continuous IHE using 3T breath-hold cine MRI-IHE. CEF (%change in CSA and CBF with IHE) comparisons were made according to age and sex, and all women ≥50 years were post-menopausal.

**Results:**

In the overall population, there were no differences in CEF between men and women. However, when stratified by age and sex the mean changes in CSA and CBF during IHE were higher in younger premenopausal women than older postmenopausal women (%CSA: 15.2±10.6% vs. 7.0±6.8%, p = 0.03 and %CBF: 59.0±37.0% vs. 30.5±24.5% p = 0.02). CBF change was also nearly two-fold better in premenopausal women than age-matched men (59.0±37.0% vs. 33.6±12.3%, p = 0.03).

**Conclusions:**

Premenopausal women have nearly two-fold better mean CEF compared to postmenopausal women. CEF, measured by CBF change is also better in premenopausal women than age-matched men but there are no sex differences in CEF after menopause. Fundamental age and sex differences in CEF exist and may contribute to differences in the development and clinical manifestations of atherosclerotic CVD, and guide future trials targeting sex-specific mechanisms of atherogenesis.

## Introduction

Despite declines in cardiovascular disease (CVD) mortality rates in the United States over the past several decades, CVD is still the leading cause of morbidity and mortality in women [1]. While premenopausal women have a low prevalence of CVD, there is a marked increase in cardiovascular risk in women after menopause [2, 3]. However, controversy exists as to whether this increased risk is due to the effect of aging alone or as a consequence of the cardio-metabolic and vascular changes that occur during menopause [4, 5]. Therefore, it is critical to better understand the mechanisms contributing to the rapid and adverse changes in CVD risk that occur as women reach menopause [6, 7] as they may guide new practical preventive and treatment approaches in this at-risk population.

Endothelial function is considered a marker of vascular health and, as such, may provide important insights into the mechanisms contributing to the development and progression of atherosclerosis with menopause [8]. Although normal endothelial function is impaired by both traditional and non-traditional CVD risk factors [9], responds favorably to risk factor modification [10], and predicts future cardiovascular events [11], studies of sex differences in endothelial function are conflicting. Some lines of research show that peripheral endothelial function measured by flow mediated vasodilation (FMD) of the brachial artery decreases with age in a gradual manner in men beginning in the fifth decade, while it remains preserved in young women followed by a more rapid decline in postmenopausal women in the sixth decade [12, 13]. However, other studies of peripheral endothelial function have not shown sex differences in systemic vasodilation and demonstrate only an age-related decline [14–17].

Impaired coronary endothelial function (CEF) plays an important role in the development of coronary atherosclerosis [18, 19], and predicts CVD events [11, 20–23]. However, it has not been well characterized in healthy populations largely due to the invasive nature of conventional approaches used to measure CEF [24]. In addition, current coronary imaging methods document anatomic coronary atherosclerosis that has developed over years, but there has not been a noninvasive means to quantify the central, early mechanisms contributing to the pathogenesis of coronary artery disease (CAD) such as coronary endothelial dysfunction. Moreover, measures of coronary and systemic endothelial function are not strongly related possibly due to differences in vascular biology between the two arterial beds [25, 26].

Recently, noninvasive measures of CEF were developed using magnetic resonance imaging (MRI). The approach measures changes in coronary artery lumen area and blood flow in response to isometric handgrip exercise (IHE), an endothelial dependent stressor [27–30]. The MRI-detected coronary responses were shown to be nitric oxide (NO)-dependent, thus indicative of CEF, and reproducible, offering a window into the pathogenesis of sex differences in atherosclerosis development [27, 30].

Despite prior invasive studies showing a high incidence of coronary endothelial dysfunction in women presenting with chest pain with non-obstructive CAD [23, 31], the role that abnormal CEF plays with regards to sex differences at earlier stages of atherosclerotic disease, particularly in asymptomatic healthy individuals is not well understood. We therefore used MRI to non-invasively quantify CEF to test the hypotheses that in healthy asymptomatic individuals with no known CAD, CEF is better in younger premenopausal women than in age-matched men and that sex differences in CEF are no longer present in older men vs. age-matched postmenopausal women.

## Materials and methods

### Participants

All participants provided written informed consent, the protocol was approved by The Johns Hopkins Medicine Institutional Review Board, and all clinical investigation was conducted according to the principles expressed in the Declaration of Helsinki. The participants were 50 healthy men and women recruited at the Johns Hopkins Hospital. The participants were defined as healthy if they were < 50 years old without a history of CAD, no more than 1 traditional cardiovascular risk factor, and a 10-year ASCVD risk estimate of less than 5% on the pooled cohort equation [32]. In addition, participants who were ≥ 50 years of age had an Agatston coronary artery calcium score of <10, or no more than mild luminal stenosis on a prior computed tomography scan [33]. Participants were excluded if they had insulin-dependent diabetes mellitus, were smokers, or were on hormone replacement therapy. All women over the age of 50 were postmenopausal defined as at least 12 months of amenorrhea [34]. No participant had a contraindication to MRI.

### Study protocol

Coronary MRI was performed using a commercial human 3.0 Tesla (T) MR scanner (Achieva, Philips, Best, NL) with a 32-element cardiac coil for signal reception. All participants were in a fasting state. Images were obtained perpendicular to a proximal, straight segment of the coronary artery best identified on double oblique scout scan as previously reported [35]. The imaging plane for the endothelial function measurements was localized in a proximal or mid coronary arterial segment that was straight over a distance of approximately 2.0 cm. In some cases, when both arteries displayed equivalent image quality, two coronary arteries per participant were imaged and the mean of the two values was used. Baseline imaging at rest for cross-sectional coronary artery area (CSA) measurements was followed by coronary flow velocity-encoded MRI using single breath-hold cine sequences [36] as previously reported [37]. The endpoints CSA, coronary flow velocity (CFV) and blood flow (CBF) were quantified before and during isometric handgrip exercise (IHE). Each participant performed continuous IHE using an MRI-compatible dynamometer (Stoelting, Wood Dale, IL, USA) for approximately 5–6 minutes at 30% of their maximum handgrip strength while under direct supervision to ensure compliance [38, 39].

Heart rate and blood pressure were measured throughout the study using a non-invasive and MRI-compatible ECG and calf blood pressure monitor (Invivo, Precess, Orlando, FL, USA). The rate pressure product (RPP) was calculated as systolic blood pressure x heart rate. Detailed MR parameters have been previously published [27]. The primary outcomes measured were percent change in CSA and CBF with IHE.

### Image analysis

Images were analyzed for coronary CSA at rest and stress using a semi-automated software tool (Cine version 3.15.17, General Electric, Milwaukee, WI, USA) by two independent readers who were blinded to the subject’s age. A circular region-of-interest was traced around the coronary artery in diastole during the period of least coronary motion, and a computer algorithm employed an automated full width half maximum algorithm for the cross-sectional coronary area measurements.

For CBF measurements, images were analyzed using commercially available software (FLOW Version 3.0, Medis, NL). Peak diastolic coronary flow velocity was used for the velocity measurements and peak diastolic coronary artery blood-flow was calculated and converted to the units mL/minute as previously reported [40].

### Statistical analysis

Statistical analysis was performed using Stata software version 14.2 (StataCorp, College Station, Texas). The average of the values was used when more than one coronary artery segment in a participant was imaged. Data were tested for normality using the Shapiro-Wilk test, and the data were normally distributed. Student’s paired *t-tests* were used to compare baseline and stress coronary artery CSA, CFV, and CBF measurements for each group. ANOVA (with Bonferroni correction for multiple groups) was used to test for between group comparisons for the primary endpoints % change in CSA, CFV and CBF with IHE, and Student’s unpaired *t-tests* were used to compare the CEF endpoints between individual groups (grouped according to sex and age). Age was treated as a binary variable with a cutoff of < 50 and ≥ 50 years. Statistical significance was defined as a two-tailed p-value <0.05. To examine whether there was a relationship between baseline CSA and %CSA change with IHE, linear regression analysis was performed between the dependent variable %CSA change and the independent variable baseline CSA. Baseline characteristics and data are expressed as mean ± standard deviation.

## Results

**Fig 1:**
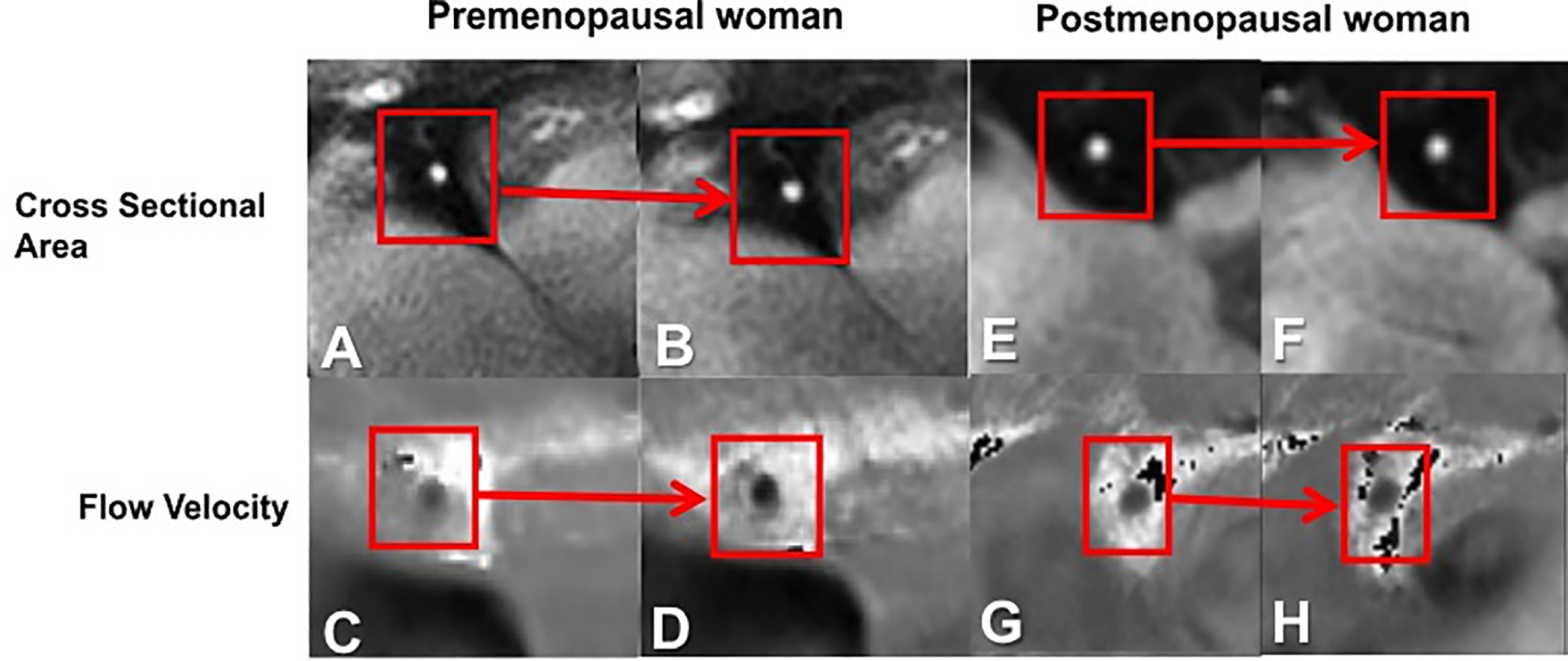
Representative coronary artery images for cross sectional area and blood velocity. Example in a healthy premenopausal woman demonstrating a right coronary artery (RCA) cross section (**A**) at rest (**B**) and during isometric handgrip exercise (IHE) showing vasodilation. Magnified flow velocity image of the RCA in the same subject is shown at rest (**C**) and during IHE (**D**) in diastole, wherein the signal phase is proportional to flow velocity with the darker pixels during IHE indicating higher velocity in the caudal direction through the RCA. Example in a healthy postmenopausal woman of the RCA cross section (**E**) at rest (**F**) and during IHE showing no vasodilation. Flow velocity image of the RCA in the same postmenopausal subject is shown at rest (**G**) and during IHE (**H**) in diastole, wherein the signal darkness does not increase during IHE as it does in the younger healthy subject.

All participants completed the MRI study. Overall there were a total of 50 participants, 20 men with a mean age 44.1 ± 16.4 years and 30 women with a mean age 49.8 ± 16.7 years. Overall women had lower estimated 10 year ASCVD risk compared to men (4.2± 2.5% vs. 8.6± 5.3% p = 0.04). The baseline characteristics of the study population are presented in Table 1. Representative coronary images for area and blood velocity are shown in Fig 1. The hemodynamic effects of isometric handgrip stress in women versus men are presented in Table 2.

**Table 1:** Baseline characteristics of the study participants.

Characteristics	Men (n = 20)	Women (n = 30)	P value
Age (years), mean (SD)	44.1 (16.4)	49.8(16.7)	0.24
<50 years, N	11	16	
≥50 years, N	9	14	
BMI kg/m^2^ mean (SD)	27.0(4.7)	27.0(5.7)	0.99
Hypertension, N (%)	3 (14)	3 (11)	0.54
Hyperlipidemia, N (%)	2 (10)	3 (11)	0.91
10 Year ASCVD risk estimate (%)	8.6 (5.3)	4.2 (2.5)	0.04
ASCVD risk, age <50 years	3 (3.5)	2.7 (1.5)	0.91
ASCVD risk, age ≥50 years	9.9 (4.8)	5.2 (2.3)	0.03
Ever smoker, N (%)	0 (0)	0 (0)	N/A
Statin, N (%)	2 (10)	3 (10)	0.91
Hormone replacement therapy, N(%)	N/A	0(0)	N/A
Oral contraceptive use, N(%)†	N/A	7(44)	N/A
Coronary segments studied			
LAD	6	6	0.44
RCA	7	17	0.14
RCA and LAD	7	7	0.39

Values are expressed as mean and standard deviation, unless otherwise specified. SD = standard deviation, BMI = body mass index, ASCVD = atherosclerotic cardiovascular disease risk score derived from pooled cohort equation of the American Heart Association and American College of Cardiology, † Oral contraceptive use in premenopausal women.

**Table 2:** Hemodynamic effect of isometric handgrip stress.

Hemodynamic variable (mean and standard deviation)	Men	Women	P value for difference
Baseline systolic blood pressure (mmHg)	129.2(16.6)	133.9(16.0)	P = 0.32
Baseline diastolic blood pressure (mmHg)	66.6(10.6)	66.6(11.6)	P = 1.00
Stress systolic blood pressure (mmHg)	143.1(19.7)	144.6(19.8)	P = 0.79
Stress diastolic blood pressure (mmHg)	77.2(13.0)	78.5(12.8)	P = 0.72
Baseline heart rate (bpm)	66.9(10.7)	66.0(8.1)	P = 0.73
Stress heart rate (bpm)	80.3(11.4)	77.4(8.3)	P = 0.32
Baseline rate pressure product (mmHg*bpm)	8602.3(1511.7)	8825.1(1510.7)	P = 0.61
Stress rate pressure product (mmHg*bpm)	11,458.2(2084.6)	11,187.2(1945.6)	P = 0.64
Rate pressure product change, % (SD)	33.9(15.3)	28.1(21.3)	P = 0.30

The values are represented as mean and standard deviation. BPM = beats per minute.

### Coronary vasodilation

**Fig 2:**
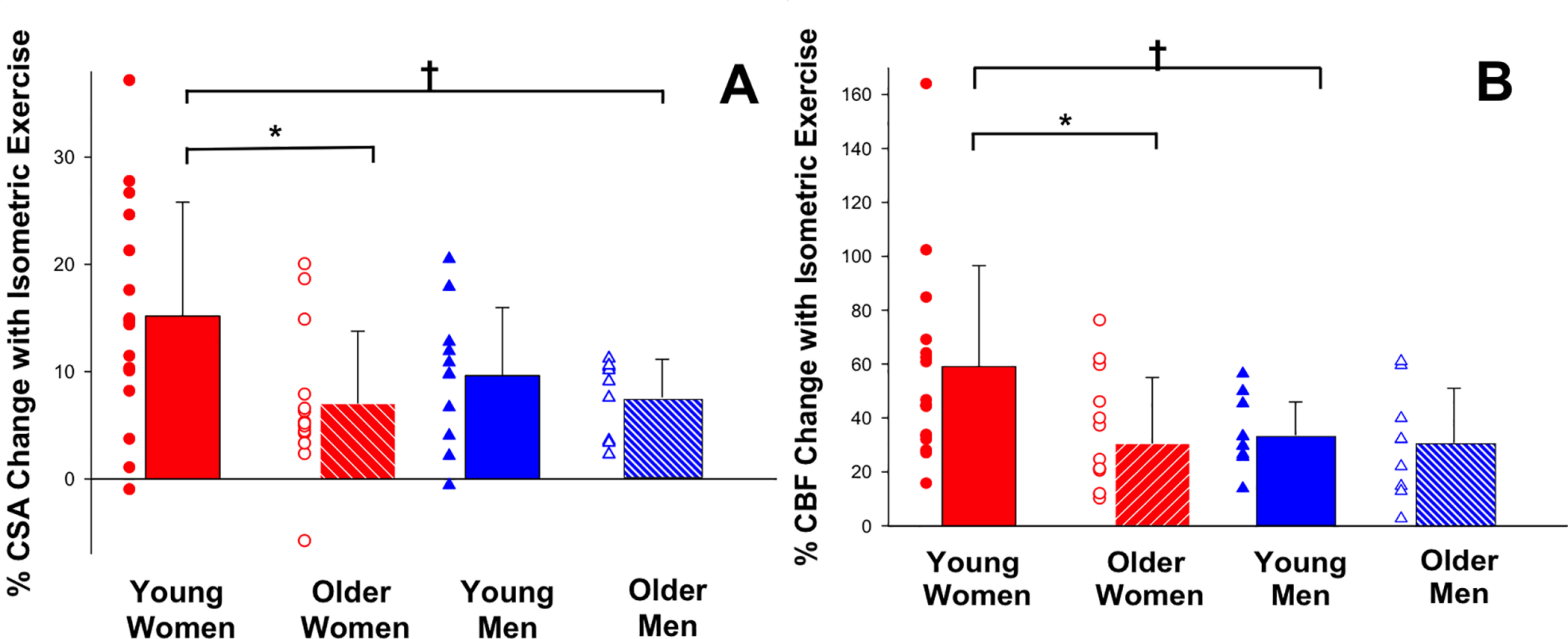
Change in coronary cross sectional area and blood flow with isometric handgrip stress (IHE). **(A)** Individual data points of relative changes in coronary vasoreactive parameters with IHE are shown for healthy subjects of both sexes (by age <50 years and ≥ 50 years) for % CSA (coronary cross sectional area) in response to IHE. Bars represent mean +/- SD. There was a significant difference in %CSA change between healthy young and healthy older women and men (* p = 0.03, †p = 0.05). **(B)** Individual data points of relative changes in coronary endothelial function are shown for healthy participants grouped by age and sex for % CBF (coronary blood flow) change in response to IHE. Bars represent mean +/- SD. The %CBF change for healthy young women was significantly higher compared to healthy older women and healthy young men (* p = 0.02 †p = 0.03).

**Fig 3:**
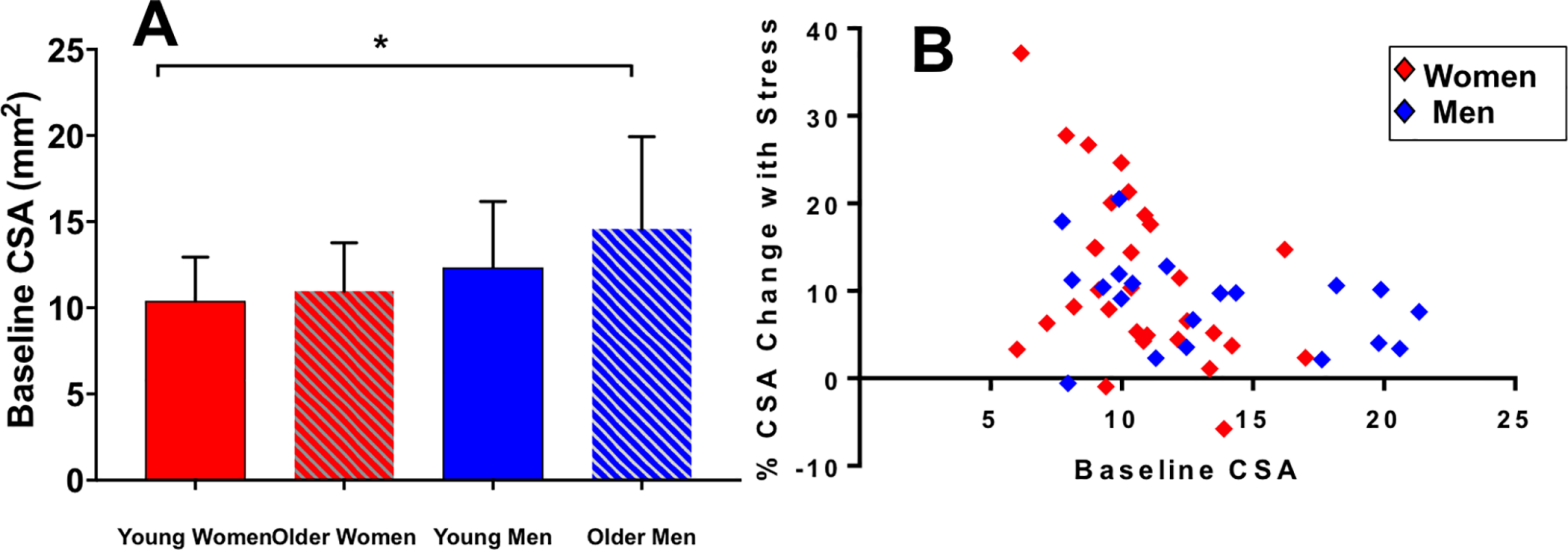
Baseline coronary cross sectional area. (A) Summary data for baseline CSA (coronary cross sectional area), showing mean +/- SD (error bars) for four groups: younger women, older women, younger men and older men. The baseline CSA is higher in older men compared to younger women (*p = 0.01). Correlation of baseline cross sectional area with percent change in cross sectional area by sex (B) There was no significant relationship between baseline CSA and % CSA change for women (red diamonds) and men (blue diamonds).

Coronary arteries dilated with IHE in both healthy men and women. In men baseline CSA was 13.4±4.6 mm^2^ and increased 8.8±5.2% with IHE (p<0.001 stress vs. baseline). In women baseline CSA was 10.7±2.6mm^2^, and increased 11.4±9.6% with IHE (p<0.001 stress vs. baseline). Men had greater CSA at baseline than women (p = 0.01). In the overall population, although %CSA and %CBF changes trended higher for women compared to men, there was no statistically significant difference in CEF between the two groups overall (p = 0.30 for %CSA change, p = 0.13 for %CBF change). However, when stratified by age (<50 and ≥50 years) and sex, the mean change in %CSA for women <50 years was 15.2±10.6%, which was significantly higher than that for women ≥50 years (7.0±6.8%, p = 0.02) and for men ≥50 years (7.6±3.6% p = 0.05, Fig 2A). Although the %CSA change in younger women (<50 years: 15.2±10.6%) trended higher than the %CSA change in age matched men (men <50: 9.6 ±6.4%), the difference was not statistically significant (p = 0.13). In the older age-matched groups (women and men ≥ 50 years), %CSA change was similar between groups (%CSA women: 7.0±6.8% vs. men: 7.6±3.6%, p = 0.6). The %CSA change in younger vs. older men was not significantly different. Younger vs. older women had similar baseline coronary area (10.4±2.6mm vs. 10.9±2.8mm, p = 0.57). Fig 3A. Using linear regression, there was no significant relationship between baseline CSA and %CSA change with IHE in the group overall (R = -0.16, p = 0.26, Fig 3B.)

### Coronary flow velocity and coronary blood flow measures

Peak diastolic coronary flow velocity increased with IHE in both men and women. In men, baseline CBF was 50.6±25.0 ml/min and increased 33.3±16.1% with IHE (p<0.001 baseline vs. stress). In women baseline CBF was 38.6±24.3 ml/min and increased 44.3±34.1% (p<0.001 baseline vs. stress). There was no significant difference in baseline resting CBF between men and women, nor among any of the four subgroups. Percent change in CBF with IHE trended higher for women than men overall, but the difference was not statistically significant. When stratified by age and sex, the mean change in %CBF for women <50 years was 59.0±37.0%, which was nearly two-fold higher than that in women ≥50 years (30.5±24.5%, Fig 2B, p = 0.02). In addition, %CBF change in younger women (<50 years) was higher than the %CBF change in age matched men (men <50 years: 33.6±12.3%, p = 0.03 vs. women<50). In contrast, in the older age-matched groups (women and men ≥ 50 years), the %CBF change was similar between groups (%CBF women: 30.5±24.5% vs. men: 30.8±20.2%, p = 0.95). There were no significant differences in %CBF change between younger and older men. Results were not different after excluding outliers that were >2 SD from the mean. Finally, the results of the primary CEF endpoints, IHE-induced %CSA and %CBF were the same when analyzed on a per-segment or per-patient basis.

## Discussion

Using noninvasive MRI measures of CEF, we observed that in a cohort of healthy individuals with no CAD, and no more than one conventional cardiovascular risk factor, young premenopausal women have two-fold better measures of CEF compared to healthy postmenopausal women. In addition, healthy postmenopausal women have measures of CEF that are comparable to men of all ages. Despite conflicting data on sex differences in peripheral vascular function with aging [12, 13], this study is the first to document sex differences in coronary endothelial function in healthy, asymptomatic individuals and shows that the coronary vasoactive NO-mediated responses are nearly two-fold higher in younger women than in older women and men. Our study complements studies of peripheral endothelial function but adds critical knowledge of the underlying fundamental differences in vascular biology between the coronary and peripheral circulation [25, 41]. Traditionally, invasive techniques have limited the assessment of CEF in low risk populations; therefore our non-invasive MRI measures of CEF in healthy, asymptomatic individuals [27] offer a unique window into the pathogenesis of sex differences in coronary vascular function.

The CEF measures reported in the current study are consistent with those previously described using non-invasive MRI measures [27, 29]. This MRI CEF technique was shown to be reproducible over the short and longer term with good inter and intra-observer variability [27, 29, 30]. Moreover, we previously showed that the NO synthase inhibitor monomethyl-L-arginine (L-NMMA) [30] prevents the normal IHE-induced increases in CSA and CBF during IHE, providing strong evidence that the coronary responses to IHE measured by MRI primarily reflect endothelial NO-mediated CEF. We now show for the first time that significant sex differences in NO-mediated coronary cross sectional area and blood flow to endothelial-dependent stressors are present in healthy individuals.

Prior invasive studies showed that sex differences exist in atherosclerotic vascular remodeling [42] and in coronary endothelial dysfunction in patients presenting with angina [31]. In the Women’s Ischemia Syndrome Evaluation (WISE) study two thirds of women referred for invasive coronary angiography for chest pain had normal coronaries or only mild non-obstructive CAD [43–45]. Moreover in individuals with early CAD referred for invasive coronary angiography, men tended to have more eccentric atheroma than women, while women had more diffuse epicardial coronary endothelial dysfunction.[45, 46] Abnormal CEF in the setting of non-obstructive CAD is strongly associated with adverse CV outcomes in women presenting with ischemia [23]. However, ours is the first study to demonstrate significant sex differences in CEF in a healthy population and that those differences are present only in younger individuals (<50 years). Our observations that measures of CEF (CSA and CBF) are better in younger women, compared to older women and CBF is better in younger women compared to age matched men are consistent with prior epidemiological observational studies showing that premenopausal women are protected from cardiovascular events compared to men of similar ages but that after menopause the sex disparities in CVD morbidity and mortality diminishes [3]. CVD remains the leading cause of mortality in women particularly after menopause, and this is hypothesized to be due to the loss of the protective effect of estrogen [47, 48]. A recent study showed that women who experience premature menopause have a higher risk of CVD mortality than do women who experience menopause later in life; therefore, the higher CV risk in women is less likely an aging effect alone [6]. Despite randomized controlled studies not showing a benefit of HRT after menopause for the primary prevention of CVD, substantial evidence has accumulated on the benefits of HRT for women who experience premature menopause, or in women who are younger than 60 years [49, 50]. In addition, primary prevention measures are rarely instituted in women of postmenopausal age who are asymptomatic and without risk factors due to underestimation of women’s risk with standard CV risk calculators [51, 52]. Importantly, the mean ASCVD risk score in our population of postmenopausal women was 5.2%, which is considered low risk. Nevertheless, CEF was impaired in these postmenopausal women compared to premenopausal women and was similar to CEF of age-matched men with a higher ASCVD risk score (9.9%). This suggests the possibility that noninvasive measures of CEF may have additive value compared to standard CV risk assessment by documenting abnormalities in CEF in selected populations who are not identified by current risk assessment tools.

Our study detected significant differences in CBF change with IHE between healthy women and men less than 50 years of age. In addition we found that younger women had higher percent change in %CSA compared to younger men however the difference was not significant possibly limited by the number of participants. Changes in CSA with IHE in general reflect macrovascular endothelial reactivity, whereas CBF change reflects both macro and microvascular reactivity. The fact that there was a larger but non- significant difference in %CSA but a significant difference in %CBF change between younger women and men suggests that both macrovascular and microvascular reactivity may play an important role in sex differences in the development of atherosclerosis in younger people. Microvascular CEF is affected by more global factors influencing downstream resistance vessels, whereas stress-induced change in CSA reflects epicardial coronary vasodilatation, which is more closely related to local factors such as anatomic remodeling and atherosclerotic disease [28]. Taken together, fundamental differences exist in coronary artery pathophysiology between men and women, even at an early stage.

Initially two-fold better blood flow-related CEF in younger women is lower in older women when compared with men after age 50, possibly due to the hormonal changes that take place during menopause [47, 53, 54], although this was not specifically tested in this study. Moreover, the significant difference in CEF observed between younger and older, postmenopausal women cannot be explained by differences in baseline coronary area, as they were very similar. Epidemiological research has shown that women generally develop CVD 10 years later than men possibly due to the protective effects of estrogen [3, 55]. The perimenopausal period may be associated with rapid adverse changes in coronary endothelial function. Menopause is generally associated with an acceleration of CV risk that begins during the perimenopausal transition [56]. Therefore, this study provides a basis for future investigation into the question of whether the noninvasive assessment of CEF is an important tool to guide more intensive evidence-based primary prevention strategies in healthy postmenopausal women who would otherwise be considered low risk on traditional risk assessment calculators [52] and studying novel therapeutic interventions to improve coronary endothelial function.[57–61]

### Study limitations

The sample size was relatively small, however it was sufficient to identify significant differences in CEF between healthy younger and older women. The non-significant trend in higher %CSA change between young men and women could have been due to the small sample size and future studies with large samples sizes may detect a difference. In addition, the groups were well-matched with regards to BMI and risk factors. Our study was observational; therefore we are unable to determine causality. In the future, a longitudinal study of CEF before and during the menopausal transition may provide more insights into the relative roles of age and menopause in reducing CEF than is possible with a cross-sectional design. A more complete understanding of the role of menopause would also be provided by studies in women with premature menopause. In addition, we did not study a broad age range of participants, which merits study in a larger population. Future studies can further examine in longitudinal fashion how quickly CEF deteriorates over time and how estrogen loss and replacement influences CEF, as the technique is safe, reproducible and therefore well suited to repeated studies in low-risk individuals.

## Conclusion

Our findings show that healthy premenopausal women who are younger than 50 years old have nearly two-fold better CEF measures than that of healthy postmenopausal women who are older than 50 years. In contrast, younger and older men have comparable CEF to that of postmenopausal women. These results are consistent with those reported previously in studies of peripheral artery endothelial function and suggest that the increase in traditional CVD risk that develops after menopause in women coincides with adverse changes in coronary endothelial function in otherwise healthy people. Our findings may also be useful for sex-specific risk assessment of asymptomatic but at risk individuals and can potentially inform larger trials aimed at examining novel primary prevention strategies.
